# Addictive Eating and Its Relation to Physical Activity and Sleep Behavior

**DOI:** 10.3390/nu10101428

**Published:** 2018-10-04

**Authors:** Jeremy Tan Ee Li, Kirrilly M. Pursey, Mitch J. Duncan, Tracy Burrows

**Affiliations:** 1 School of Health Sciences, Faculty of Health and Medicine, The University of Newcastle, Callaghan, NSW 2308, Australia; jeremy.tan.e.l@skh.com.sg; 2 Department of Dietetics, Sengkang General Hospital, Singapore Health Services, Singapore 544886, Singapore; 3 Priority Research Centre for Physical Activity and Nutrition, The University of Newcastle, University Drive, Callaghan, NSW 2308, Australia; Kirrilly.Pursey@newcastle.edu.au (K.M.P.); Mitch.duncan@newcastle.edu.au (M.J.D.); 4 School of Medicine & Public Health, Faculty of Health and Medicine, The University of Newcastle, University Drive, Callaghan, NSW 2308, Australia

**Keywords:** food addiction, obesity, Yale Food Addiction Scale, physical activity, sedentary behavior, sleep behaviors, sleep duration, sleep quality

## Abstract

The obesity epidemic has led to the exploration of factors contributing to its etiology. Addictive eating, physical activity, and sleep behaviors have all been independently associated with obesity, and recent research suggests plausible interrelationships between food addiction, physical activity, and sleep. This study aims to investigate the relationship between food addiction with physical activity and sleep behavior. Australian adults were invited to complete an online survey which collected information including: demographics, food addiction symptoms, physical activity, sitting time and sleep behavior items. The sample comprised 1344 individuals with a mean age of 39.8 ± 13.1 years (range 18–91), of which 75.7% were female. Twenty-two percent of the sample met the criteria for a diagnosis of food addiction as per the Yale Food Addiction Scale (YFAS 2.0) criteria, consisting of 0.7% with a “mild” addiction, 2.6% “moderate”, and 18.9% classified as having a “severe” food addiction. Food-addicted individuals had significantly less physical activity (1.8 less occasions walking/week, 32 min less walking/week, 58 min less moderate to vigorous physical activity (MVPA)/week; *p* < 0.05), reported sitting for longer on weekends (83 min more on weekends/week; *p* < 0.001), and reported significantly more symptoms of poorer-quality sleep (more likely to snore, more likely to have fallen asleep while driving, reported more days of daytime falling asleep; *p* < 0.05) compared to non-food-addicted individuals. These differences were also observed in those with a “severe” food addiction classification. The present study suggests frequency and duration of physical activity, time spent sitting and sleep duration are associated with food addiction.

## 1. Introduction

Obesity is one of the most significant global public health issues of the 21st century [1]. The World Health Organisation (WHO) estimated that as of 2014, 39% (over 1.9 billion) of the world’s adult population was overweight and 13% (600 million) were obese [2]. Similarly, a systematic review found that from 1980 to 2013, the global prevalence of overweight and obesity increased 27.5% for adults and 47.1% for children [1].

The prevalence of obesity and the health and socioeconomic burdens that accompany it [3,4] have prompted extensive research into its possible causes. Genetic susceptibility may be a non-modifiable risk-factor for obesity [5,6]; however, there are a multitude of modifiable risk factors involved. Diet influences energy intake, while physical activity is a modifiable aspect of energy expenditure [7]. As such, dietary choices [8], frequency and duration of physical activity and sedentary behaviors, and an increasingly obesogenic environment [9] are considered major factors contributing to energy imbalance. Increasingly, psychological factors are being investigated in the etiology of obesity [10,11]. Studies have found similarities in personality types, behavioral traits, and functional neural abnormalities between obese individuals and those with addictive disorders such as drug or alcohol dependence [12,13,14]. This has led to an increased amount of research investigating that an addictive process in relation to food and/or eating—“food addiction”—may contribute to the development of obesity in susceptible individuals.

“Food addiction” is a debatable topic—not least because it currently has no standardized definition and terminology often debated [15], with terminology such as “eating addiction” and “food use disorder” suggested [16,17]. Tools have been developed to measure food addiction, including the Yale Food Addiction Scale (YFAS) [18], which is a validated self-administered survey where criteria directly relate to the Diagnostic and Statistical Manual of Mental Disorders (DSM) criteria for substance use disorders [18,19]. The term “food addiction” will be used in this study. Systematic reviews which included both clinical and population groups have identified an average prevalence of food addiction between 15 and 20% [20,21] with higher prevalence often found in clinical population groups such as those with binge eating disorder. Higher YFAS symptom scores have been found to be associated with higher rates of obesity [18], personality characteristics and higher intakes of energy dense foods [22]. Food addiction is not synonymous with obesity as food addiction can also occur in healthy weight individuals. 

Physical activity has long been implicated in the etiology of obesity. Worldwide, physical activity levels appear to be decreasing: technological advancements have led to a steady decline in both occupational and household activity/chore related physical activity, with an increasing proportion of the global population employed in sedentary jobs and engaging in less household physical activity [7,23,24]. Furthermore, a significant proportion of the global population appears to not meet physical activity guidelines [25]. Insufficient physical activity levels combined with increased sedentary behaviors, (defined as energy expenditure <1.5 METs while sitting or reclining [26]) decreased energy expenditure and may place an individual closer to the state of positive energy balance required for the development of obesity. Smaller increases in Body Mass Index (BMI) and waist circumference were observed in individuals with high physical activity levels compared to those with low physical activity levels in longitudinal studies over 20–35 years [27,28]. Beyond its effect on BMI and weight status, physical activity may also have a role in the regulation of appetite [7]. Shook et al. [29] found adults with the lowest physical activity levels reported higher levels of both food cravings and disinhibition when compared to those with the highest physical activity levels, in addition to a higher body weight and one year fat mass gain [29]. Cravings and disinhibition are implicated in addictive behavior [19], suggesting that a link may exist between food addiction and physical activity—which might further elucidate the observed link between physical activity and obesity.

Evidence suggests a two-way relationship exists between diet and sleep. Firstly, studies and reviews have found that dietary components including vegetable, fish, caffeine, and confectionary intake [30], the type, amount and glycemic index (GI) of carbohydrates [31], the tryptophan quantity of foods [32], as well as behaviors such as skipping breakfast [30], have the potential to influence both the duration and quality of sleep [32,33,34]. Daytime dysfunction such as unintentionally falling asleep (including while driving) may be reflective of poor quality and/or short sleep duration [35], while sleep disordered breathing, including snoring, can negatively impact sleep quality [36]. Short sleep duration as well as long sleep duration may be linked to the development of obesity through its association with unhealthy eating patterns including increased intakes of energy and fat, increased ratio of fat to protein intake [37,38], and excessive calorie consumption from snacks [39,40,41,42,43]. Insufficient sleep may negatively affect regulation of food intake, making unhealthy food choices more appealing and rewarding [44]. Several functional Magnetic Resonance Imaging (fMRI) studies have shown increased activation in brain regions associated with food behaviors and pleasure/reward-seeking during periods of sleep restriction or deprivation [45,46,47,48], especially in response to unhealthy, high caloric foods [45]. Interestingly, similar patterns of neural activation have been observed in individuals with food addiction [49], which raises the possibility of a relationship between sleep behaviors and food addiction. If such a relationship does exist, it may form an important intermediary step in the observed association between sleep behavior and the development of obesity. A bidirectional relationship has also been identified between sleep and physical activity, with poor sleep contributing to lower physical activity [50], and physical activity associated with improved sleep duration and quality [51].

Few studies have investigated associations between food addiction, physical activity and sleep; those which do exist have been limited to specific population groups including college students only [52], or have not assessed associations with food addiction severity [53]. Existing studies show that high levels and not intensity of physical activity may be associated with more symptoms of food addiction [52] and food addiction related to poorer sleep quality [53]. Further research into the interrelationships between addictive-like eating, physical activity and sleep in a broader sample is warranted. The aims of this study are to investigate if food addiction diagnosis and severity, as measured using YFAS 2.0, is associated with physical activity and/or sleep behaviors. It is hypothesized that individuals with food addiction diagnosis and a higher level of severity would have lower physical activity levels—with less physical activity and higher sedentary behavior. It is also hypothesized that individuals with a food addiction diagnosis and higher level of severity will have shorter sleep duration, and poorer-quality sleep than those without food addiction.

## 2. Materials and Methods

### 2.1. Participants

The present study is a secondary data analysis as part of a larger study investigating addictive eating behaviors and mental health [22]. Data for the current analysis was collected via an online survey, which was conducted from April to July 2016. In brief, participants were recruited to complete the survey through University of Newcastle media releases, and social media platforms such as Facebook and Twitter. These advertisements contained a link through which participants could access the online survey, which took approximately 20 min to complete. Participants were initially screened for eligibility with three questions at the beginning of the survey. Inclusion criteria for the study were: participants aged ≥18 years, living in Australia, and able to read and understand English. Pregnant and lactating women, as well as those unable to understand English, were excluded from the study. As an incentive, survey completers were offered the chance to win one of 10 × $50 shopping vouchers via a random prize draw. Full ethics approval was obtained from the University of Newcastle Human Research Ethics Committee (HREC; H-2016-0010).

### 2.2. Measures

The survey included a total of 138 self-reported items, including: demographics, food addiction as measured by the YFAS 2.0 [18], physical activity using items from the Active Australia Survey [54], sitting and sleep behaviors were assessed, presence of binge eating symptoms as measured using the Binge Eating Scale (BES) [55], presence of depression, anxiety and stress as measured by the Depression Anxiety and Stress Scale (DASS) [56], personality dimensions as measured by the Substance Use Risk Profile Scale (SURPS) [57], and dietary intake as measured using 20 standardized questions adapted from the New South Wales Health Survey [58]. As part of the current study, only variables relating to demographics, food addiction, physical activity, and sleep behaviors were analyzed, while outcomes related to personality phenotypes, mental health and dietary intake in Australian adults with and without food addiction have previously been reported [22].

*Demographics*: Nine items were collected, including gender, age, ethnicity, marital status, highest level of education, and postcode. Postcode was used to assess participants area level socioeconomic status (SES) using the Index of Relative Socioeconomic Advantage and Disadvantage (IRSAD) which classifies postcodes into deciles from one (most disadvantaged/least advantaged) to 10 (least disadvantaged/most advantaged) [59]. Participants were also asked to self-report their body measurements, including current height (cm) and weight (kg). BMI kg/m² was calculated using standard equations and interpreted using the WHO BMI cut offs. 

*Food addiction*: This was assessed using the YFAS 2.0 [18], which is an updated version of the original YFAS. The revised tool reflects changes in the DSM-V criteria for substance use disorders [19], and has been validated for use in both children and adults, with appropriate convergent and divergent validity, as well as internal consistency [60,61]. The YFAS 2.0 contains 35 questions corresponding to 11 DSM-V symptom criteria for Substance-Related and Addictive Disorders (SRAD) [18,19]. Each question comprises of eight possible responses ranging from “never” to “every day”. A symptom was considered met if one or more of the questions associated with the criteria meet a pre-defined threshold [18]. The YFAS 2.0 provides a “diagnosis” of food addiction (FA), which is classified according to “severity” based on the number of symptoms endorsed. A mild severity is allocated when two to three symptoms present, moderate when four to five symptoms are present, or severe when six or more symptoms are present. The YFAS 2.0 tool asks individuals to consider particular behaviors to specific foods such as those high in fat and refined carbohydrates [18]. As this study also assessed overall dietary intake, to avoid priming the language used in the YFAS 2.0 questionnaire was modified for the current survey and replaced with “all foods”.

*Physical activity*: Six items from Active Australia Survey [54] were used to assess the duration and frequency of walking, moderate and vigorous intensity physical activity over the previous week. Moderate and vigorous intensity physical activities were combined when examining the frequency and duration of these activities. Four physical activity outcomes are reported in the current study: frequency of walking, frequency of moderate to vigorous intensity physical activity (MVPA), duration of walking, duration of MVPA.

*Sitting behaviors:* The total time spent sitting on a weekday, and weekend day was also assessed using the two items, this was expressed as hours and min/day with the recall period of the last 7 days. 

*Sleep behaviors*: Four questions were used to assess aspects of sleep behavior, Sleep duration was assessed by asking participants about their average daily hours of sleep in a 24-h period (to the nearest hour). Indicators of subjective sleep quality was assessed by asking participants in the last 30 days: (1) the number of days in the past month where they had unintentionally fallen asleep during daytime, (2) if they snored, and (3) if there were any occasions of falling asleep while driving in the past month.

### 2.3. Statistical Analysis

Prior to conducting statistical analysis, raw data from the survey was checked for normality and any outliers. Statistical Package for the Social Sciences (SPSS) software was then used to conduct descriptive statistics on study variables. For categorical variables, Chi-square tests were used to test for differences between groups.

For continuous variables including occasions and duration of walking and MVPA, time spent sitting and average sleep duration, the Shapiro-Wilk test was first used to determine if data was normally distributed. As the data for these variables was not normally distributed, non-parametric tests (Mann-Witney U, Kruskal Wallis) were used in the analysis of continuous data. A missing value analysis was undertaken, and the demographics of those with missing values were compared, with no patterns identified [22]. Data was analyzed according to two main categories, food addiction diagnosis (FA diagnosis—non-food-addicted and food-addicted), and food addiction severity (FA severity). As overall there were low numbers in the “mild” and “moderate” groups, these were combined, resulting in three groups for FA severity: “none” (non-food-addicted), “mild/moderate”, and “severe”. For both FA diagnosis and FA severity categories, post-hoc Kruskal Wallis tests were conducted out according to subgroups: gender (Male/Female), and BMI category (healthy/underweight, overweight, obese). Effect sizes were calculated using Cohens D.

Multiple linear regression was conducted to develop a model for predictors of FA symptoms (0–11). A range of variables were tested in univariate models (*n* = 11); however, only variables which were significant predictors (*p* < 0.05) were retained in the model. These variables retained in the analysis as covariates were BMI, duration of MVPA, time spent sitting on weekends, and number of days of unintentional daytime falling asleep in the past month (*n* = 4). Variables that were tested but excluded were occasions walking, occasions of vigorous physical activity, weekday sitting, total sitting time, total physical activity time, total occasions of weekly physical activity, and average sleep (*n* = 7).

## 3. Results 

### 3.1. Demographics

The study sample consisted of 1344 individuals, with a mean age of 39.8 ± 13.1 years (range 18–91). Forty-five percent (*n* = 605) were in the 18–35-year age group, and 75.7% were female (*n* = 1017). Only 2% of participants (*n* = 29) identified as indigenous. The majority of the sample was married (*n* = 595, 44.3%), a further 364 individuals (27.1%) were never married, 195 individuals (14.5%) were de-facto and the rest were either divorced, separated, or widowed.

Approximately 22% (*n* = 228) of the sample met the criteria for a diagnosis of FA. Of these, 34 individuals (3.3%) were classified as having a “mild” (0.7%) or “moderate” (2.6%) FA, while 194 individuals (18.9%) were classified as having a “severe” FA. The internal consistency of the YFAS was Cronbach alpha α = 0.95.

A higher proportion of females were food-addicted compared to males (*p* = 0.001), while food-addicted individuals had a lower mean IRSAD compared to non-food-addicted individuals (*p* < 0.001; Table 1). Mean BMI was significantly higher in food-addicted individuals than non-food-addicted individuals (*p* < 0.001); the obese BMI category had the greatest proportion of food-addicted individuals, followed by the overweight category, with the healthy/underweight category having the lowest proportion of food-addicted individuals. These differences were also observed for FA severity, with differences being most pronounced between the “none” and “severe” groups (Table 1; *p* < 0.05).

### 3.2. Physical Activity 

For measures of physical activity, food-addicted individuals had significantly lower frequency of walking (mean ± SD 4.9 ± 5.7 vs. 6.8 ± 5.6 occasions, <0.001), and duration of walking (160.2 ± 195.0 vs. 191.7 ± 183.8 min, <0.001), as compared to non-food-addicted individuals. There were no differences between food-addicted and non-food-addicted individuals in frequency of moderate activity (0.9 ± 1.7 vs. 1.0 ± 2.1 occasions, *p* = 0.28). Food-addicted individuals reported lower duration of MVPA as compared to non-food-addicted individuals (113.1 ± 155.5 vs. 171.2 ± 208.6 min, *p* < 0.001). Similar results were found when physical activity variables were examined by FA severity (Table 2). Individuals with a “severe” FA reported engaging in significantly lower frequency and duration of walking when compared to non-food-addicted individuals (*p* < 0.001). Individuals with a “severe” FA also reported significantly less frequently engaging in vigorous physical activity and spent significantly less time engaged in MVPA when compared to both individuals with a “mild/moderate” FA, and non-food-addicted individuals (*p* < 0.001). The “mild/moderate” FA group displayed better physical activity (PA) behaviors (duration of walking and MVPA) compared to non-food-addicted individuals however these differences were not statistically significant. The effect sizes for all PA measures were deemed small (Cohens d < 0.3).

For measures of sitting time, food-addicted individuals spent significantly more time sitting over the weekend as compared to non-food-addicted individuals (463.8 ± 302.5 vs. 381.3 ± 292.8 min, *p* < 0.001). There were no significant differences in weekday sitting between food-addicted and non-food-addicted individuals (*p* = 0.27). Similar results were obtained when analysis was carried out according to FA severity; individuals with a “severe” food addiction spent significantly more time sitting on the weekend compared to non-food-addicted individuals (Table 2).

### 3.3. Sleep

In terms of sleep duration, food-addicted individuals reported spending less time sleeping, a small but statistically significant difference when compared to non-food-addicted individuals (7.1 ± 1.4 vs. 7.3 ± 1.0 h, *p* = 0.003). No significant differences were found in the reported sleep duration between individuals based on FA severity (*p* = 0.08; Table 3).

For measures of sleep quality, food-addicted individuals were significantly more likely to report snoring compared to non-food-addicted individuals (43.8% vs. 32.4%, *p* = 0.01). Food-addicted individuals were significantly more likely to report having fallen asleep while driving in the past 30 days compared to non-food-addicted individuals (6.2% vs. 3.2%, *p* = 0.01), and reported significantly more days of unintentional daytime falling asleep in the past month compared to non-food-addicted individuals (6.1 ± 7.4 vs. 3.8 ± 5.0 days, <0.001). When these variables were examined by FA severity, individuals with a “severe” food addiction (47.5%) were significantly more likely to report snoring compared to those with a “mild/moderate” food addiction (24.2%) and non-food-addicted individuals (32.4%, *p* = 0.002). Individuals with a “severe” (6.2%) or “mild/moderate” FA (6.1%) were more likely to report falling asleep while driving compared to non-food-addicted individuals (3.2%, *p* = 0.01). Individuals with a “severe” FA reported almost twice as many days of daytime falling asleep in the past month compared to non-food-addicted individuals (*p* < 0.001; Table 3).

### 3.4. Regression Analysis 

The variables included in the multiple regression model significantly predicted the number of FA symptoms (0–11), *F*(4, 435) = 46.8, <0.001, *R*^2^ = 0.29. BMI, time spent sitting on weekends, and days of unintentional daytime falling asleep were significant positive predictors of FA symptoms, while weekly time spent on MVPA was a significant negative predictor. For every 1 kg/m^2^ increase in BMI, FA symptoms increased by 0.19 ± 0.018 symptoms (95% 329 CI: 0.154–0.226 *p* < 0.001). For each additional minute spent sitting on weekdays, FA symptoms increased by 0.001 ± 0.0 symptoms (95% CI: 0.000–0.002, *p* = 0.057). For each additional day of unintentional daytime falling asleep per month, FA symptoms increased by 0.67 ± 0.021 symptoms (95% CI: 0.026–0.107, *p* = 0.001). For each minute increase in weekly MVPA, FA symptoms decreased by 0.002 ± 0.001 symptoms (95% CI: −0.003–−0.001, *p* = 0.002).

## 4. Discussion

The present study aimed to examine potential associations between FA, physical activity, sitting and sleep behavior including both duration and quality of sleep. Overall, the prevalence of FA in the sample was 22.2%, with women significantly more likely to meet diagnostic criteria for FA than men (*p* = 0.001), and obese individuals significantly more likely to meet these same diagnostic criteria than overweight and healthy/underweight individuals. These findings are similar to previous research [20]. With regards to severity, most individuals in this sample who were food-addicted met the diagnostic criteria for a “severe” FA (18.9%), with much fewer individuals classified as “mild” or “moderate” (3.3% combined). These findings are similar to two previous studies that used the YFAS 2.0 tool, which found that “severe” FA was far more commonly diagnosed than other severity classifications [18].

Individuals classified as food-addicted as defined by YFAS 2.0 [18] were found to spend less time engaged in physical activity and more time sitting on weekends when compared to non-food-addicted individuals. The differences in MVPA and sitting time between food-addicted and non-food-addicted individuals suggest that food-addicted individuals were less active overall, engaging in less MVPA and more sitting time. However, the magnitude of differences between groups in frequency of vigorous physical activity was small. These findings do have to be interpreted with caution as self-reported measures of physical activity have been found to overestimate MVPA in adults compared to objective measures such as accelerometers [62], and there is also recent evidence suggesting that the accuracy of self-reported physical activity varies with weight status, with obese individuals most likely to over report time spent exercising compared to normal weight individuals [62,63,64]. As such, the use of self-reported measures of physical activity may have influenced the results and is a limitation of the current study.

It is noted that the mild/moderate FA group displayed better PA behaviors than the non-addicted group however these were not statistically significant. This may suggest that the mild/moderate FA group may be engaging is some compensatory behaviors for addictive over eating such as physical activity than the severe FA group. Low physical activity levels may lead to increased food cravings and disinhibition [29], which may contribute towards the addictive process [19]. Similarly, physical activity may have the potential to serve as a treatment for conventional substance use disorders (e.g., alcohol, nicotine, drugs) by potentially reducing cravings [65], withdrawal symptoms, anxiety and depression, and increasing abstinence [66]. There is also some evidence suggesting that greater sitting time and other sedentary behaviors in addition to sleep disorders may also influence mental health outcomes [67] given sleep disorders can be a core symptom of depression [68]. Given the YFAS 2.0 tool uses DSM-V criteria for substance use disorders to categorize FA [18,19], the aforementioned mechanisms may provide direction for future research examining the potential of physical activity as a treatment for FA. People engage in many patterns of activity and sitting, and people can engage in both higher levels of MVPA and higher levels of sitting. Consequently, it will be useful to for future research to examine both activity and sitting behaviors using more robust study designs to better understand the interaction between these behaviors and FA, including examining the potential of physical activity in the treatment of FA. 

Differences were identified in sleep duration between food-addicted and non-food-addicted individuals; however, these equated to approximately only 10 min less sleep for food-addicted individuals. It should be acknowledged self-report and objective measures of sleep duration do not align and this may have also influenced the differences in sleep duration observed [69]. However, food-addicted individuals and those with more severe addiction severity had higher proportions of people who snored, unintentionally fell asleep and fell asleep while driving relative to non-food-addicted or those with less severe symptoms. This suggests that their overall sleep quality is likely poorer, which given the links between appetite regulation and sleep deprivation may partly explain this finding. This is consistent with results from the regression analysis, which showed symptoms of poorer sleep quality to be a significant positive predictor of FA symptoms (*r* = 0.548, *p* = 0.001). These findings suggest that in individuals who meet sleep recommendations (such as those in this sample), sleep quality may be a more important contributing factor associated with FA than sleep duration. Unintentionally falling asleep may also be an indicator of a sleep disorder and the presence of a sleep disorder could not be established in the current study. Moreover, a recent study found that elevated YFAS scores were correlated with more symptoms of Night Eating Syndrome (NES), and were associated with poorer sleep quality [53]. As such, future research is needed to establish the effect of sleep quality on FA symptoms, including the possible mechanisms responsible, in individuals who meet recommendations for sleep. Healthy sleep is multicomponent in nature [69] and future studies examining the role of sleep in FA are encouraged to examine FA in comparison to the multiple components of health sleep to better understand this relationship. It is also noted the positive benefits that physical activity and sleep have on mental health in addition to weight status and eating behaviors [70].

This study does have several limitations. Firstly, a cross-sectional data collection method was used, which means that findings cannot be used to determine causality, such as the direction of the associations found between FA and physical activity, and FA and sleep. Further research using longitudinal study design rather than cross-sectional design could further examine how changes in FA and weight status influence sleep. Another limitation is the use of self-reported measures. In addition to limitations specific to self-reported measures of physical activity and sleep discussed earlier, recall-bias also has the potential to introduce inaccuracies to self-reported data. Physical activity items assessing the duration of moderate and vigorous activity were combined and have the potential to affect results. The domain of sitting (i.e., work /home) was not specified and the sleep measures were not categorized by weekday or weekend. Additionally, generalizability of findings is limited by sample characteristics. Firstly, the Australian sample has a unique food environment that cannot automatically be transferred to other contexts, as rates of FA vary widely by geographic region [20]. Secondly, the high number of females (75.7%) is not representative of the general population. Similarly, comparisons by categories of FA severity may be less reliable due to low numbers of individuals with “mild” and “moderate” FA (*n* = 34) as compared to individuals with “severe” FA (*n* = 194). Sub-group analysis by BMI category yielded inconsistent results, and no meaningful conclusions could be drawn regarding differences in physical activity and sleep between food-addicted and non-food-addicted individuals within the various BMI categories. People with dietary intolerances, activity limitations and sleep disorders were not screened and excluded from the study. The results of this study indicate interventions targeting those who report addictive-like overeating should consider increase PA and ensure adequate sleep is obtained in addition to dietary changes.

Despite these limitations, the present study comprised a relatively large sample size, used a validated tool (YFAS 2.0 [18]) for the assessment of FA, and examined novel outcome measures (PA, sleep) and their association with addictive eating behaviors. The present study has demonstrated cross-sectional associations between FA and both physical activity and sleep quality. Further experimental research is needed to validate these findings, address the limitations of this study, and explore the existence of potential underlying mechanisms. Future studies should consider the use of more detailed and comprehensive measures of physical activity and sleep. Objective measures of PA such as accelerometers, more comprehensive measures assessing the multicomponents of healthy sleep and/or objective measures of sleep quality/duration—such as polysomnography (PSG) or actigraphy [71], will provide more accurate physical activity and sleep data. In addition, studies controlling for equal numbers of male and female participants, and equal numbers of participants for each category of FA severity, will facilitate more meaningful comparisons between these groups. Additionally, more research is needed to understand FA in indigenous individuals. 

## 5. Conclusions

Overall, this study demonstrated an association between severe FA and lower frequency and duration of physical activity and more time sitting. Additionally, the presence of FA was also associated with poorer sleep quality. Future research to investigate the underlying mechanisms for these observations is warranted and will add to our understanding of FA.

## Figures and Tables

**Table 1:** Demographics of participants by Food Addiction diagnosis and Food Addiction severity.

Test Category	Food Addiction Diagnosis			Food Addiction Severity			
Characteristic	Non-Food-Addicted, *n* (%)	Food-Addicted, *n* (%)	*p* Value ^1^	None	Mild/Moderate	Severe	*p* Value ^2^
**Overall**	800 (77.8%)	228 (22.2%)	-	800 (77.8%)	34 (3.3%)	194 (18.9%)	-
**Gender**							
** - Male**	176 (86.7%)	27 (13.3%)	0.001	176 (86.7%)	4 (2%)	23 (11.3%)	0.003
** - Female**	624 (75.6%)	201 (24.4%)		624 (75.6%)	30 (3.6%)	171 (20.7%)	
**Age group**							
** - 18–35 years**	357 (78.6%)	97 (21.4%)		357 (78.6%)	15 (3.3%)	82 (18.1%)	
** - 35–55 years**	314 (77.7%)	90 (22.3%)	0.761	314 (77.7%)	18 (4.5%)	72 (17.8%)	0.01
** - 55+ years**	129 (75.9%)	41 (24.1%)		129 (75.9%)	1 (0.6%)	40 (23.5%)	
**IRSAD ^3^**	6.685	6.03	<0.001	6.68 ^a^	6.7 ^a,b^	5.91 ^b^	0.001
**Mean BMI ^4^ (kg/m^2^)**	25.9	32.2	<0.001	25.9 ^c^	29.1 ^d^	32.8 ^d^	<0.001
**BMI category ^5^**							
** - Healthy/underweight**	427 (92%)	38 (8%)		427 (91.8%)	12 (2.6%)	26 (5.6%)	<0.001
** - Overweight**	208 (78.5%)	57 (21.5%)	<0.001	208 (78.5%)	10 (3.8%)	47 (17.7%)	
** - Obese**	158 (54.7%)	131 (45.3%)		158 (54.7%)	12 (4.2%)	119 (41.2%)	

^1^ Between group differences for demographic characteristics were assessed using Chi-square tests (*p* < 0.05 considered statistically significant). ^2^ Differences in food addiction severity according to demographic characteristics were assessed by Kruskal Wallis tests (*p* < 0.05). ^a,b,c,d^ Mean values that do not share superscripts are significantly different from each other: results of post-hoc Kruskal Wallis tests (*p* < 0.05). ^3^ SES assessed by IRSAD scale (1–10; 1 = most disadvantaged, 10 = least disadvantaged); ^4^ Body Mass Index; ^5^ Underweight < 18.5 kg/m^2^, Healthy: 18.5–24.9 kg/m^2^, Overweight: 25–29.9 kg/m^2^, Obese > 30 kg/m^2^.

**Table 2:** Physical Activity variables by Food Addiction severity.

Variable	Food Addiction Severity, (*n*)	Mean (*n*) ± Std. Dev	*p* Value ^1^
**Frequency of walking**	None (671) ^a^	6.8 ± 5.6	<0.001
	Mild/Moderate (31) ^a,b^	7.0 ± 6.8	
	Severe (174) ^b^	4.6 ± 5.4	
	Total (876)	6.3 ± 5.6	
**Duration of walking (min)**	None (672) ^a^	191.7 ± 183.8	<0.001
	Mild/Moderate (32) ^a,b^	208.9 ± 205.5	
	Severe (175) ^b^	151.3 ± 192.3	
	Total (879)	184.3 ± 186.9	
**Frequency of vigorous physical activity**	None (673) ^a^	2.1 ± 2.4	<0.001
	Mild/Moderate (31) ^a^	3.2 ± 3.2	
	Severe (171) ^b^	1.9 ± 3.7	
	Total (875)	2.1± 2.8	
**Frequency of moderate physical activity**	None (621) ^a^	1.0 ± 2.1	0.38
	Mild/Moderate (30) ^a^	0.7 ± 0.8	
	Severe (161) ^a^	0.9± 1.9	
	Total (812)	1.0 ± 2.0	
**Duration MVPA (min)**	None (633) ^a^	171.2 ± 208.6	<0.001
	Mild/Moderate (31) ^a^	193.6 ± 213.9	
	Severe (158) ^b^	97.3 ± 136.7	
	Total (822)	157.9 ± 199.1	
**Time spent sitting on weekdays (min)**	None (674) ^a^	829.9 ± 813.7	0.10
	Mild/Moderate (31) ^a^	700.7 ± 824.2	
	Severe (171) ^a^	915.5 ± 917.7	
	Total (876)	842.1 ± 835.5	
**Time spent sitting on weekends (min)**	None (675) ^a^	381.3 ± 292.8	<0.001
	Mild/Moderate (32) ^a,b^	390.9 ± 272.6	
	Severe (171) ^b^	477.5 ± 306.6	
	Total (878)	400.4 ± 297.0	

^1^ Between group differences for physical activity were assessed by Kruskal Wallis tests (*p* < 0.05). ^a,b^ Mean values that do not share superscripts are significantly different from each other: results of post-hoc Kruskal Wallis tests (*p* < 0.05).

**Table 3:** Sleep variables (numerical) by Food Addiction severity.

Variable	FA Diagnosis	Mean (*n*) ± Std. Dev	*p* Value ^1^
Average sleep duration (hours)	None (681) ^a^	7.3 ± 1.0	0.08
	Mild/Moderate (33) ^a^	7.2 ± 1.0	
	Severe (175) ^a^	7.1 ± 1.5	
	Total (889)	7.3 ± 1.1	
Unintentional daytime falling asleep (days past month)	None (353) ^a^	3.8 ± 5.0	<0.001
	Mild/Moderate (16) ^a,b^	3.4 ± 4.1	
	Severe (116) ^b^	6.4 ± 7.7	
	Total (485)	4.4 ± 5.8	

^1^ Between group differences for sleep variables were assessed by Kruskal Wallis tests (*p* < 0.05). ^a,b^ Mean values that do not share superscripts are significantly different from each other: results of post-hoc Kruskal Wallis tests (*p* < 0.05).
